# Supplementary material for: The Role and Mechanism of Retinol and Its Transformation Product, Retinoic Acid, in Modulating Oxidative Stress-Induced Damage to the Duck Intestinal Epithelial Barrier In Vitro
Source: Animals (Basel). 2023 Oct 4;13(19):3098. doi: 10.3390/ani13193098 (PMC10572057; doi:10.3390/ani13193098)
Supplement: Supplementary file 1 [file animals-13-03098-s001.zip › animals-2601165-supplementary.pdf]

Table S1. The top twenty up-regulated and down-regulated genes between TG1 and TG2.

Gene ID	Gene name	Fold change	P value	
101792211	CHAC1	1.178537534	2.58E-52	up
110353991	HSPB8	1.344273343	1.85E-37	up
113845644	SSBP4	1.116581346	9.20E-35	up
101797711	FSTL3	1.434329473	2.71E-16	up
101798011	LOC101798011	1.493713924	1.41E-13	up
101799332	ASTN2	1.535084133	1.71E-13	up
101795265	THSD7A	1.183965182	3.62E-13	up
novel.73	-	1.256177493	5.49E-09	up
113841346	LOC113841346	4.462997465	1.86E-08	up
113845645	MEF2B	1.135626402	4.51E-07	up
113845197	LOC113845197	1.037309093	1.07E-06	up
113840243	LOC113840243	1.13335588	4.57E-06	up
101802303	PTGR1	1.110277313	4.59E-06	up
101802086	AQP9	1.533984887	9.69E-06	up
novel.361	-	1.270819003	1.01E-05	up
110353529	LOC110353529	1.701961579	1.53E-05	up
106020662	LOC106020662	1.9998328	4.57E-05	up
113845267	LOC113845267	1.003551396	5.42E-05	up
101802316	NDUFA4L2	1.083133384	6.29E-05	up
106016573	LOC106016573	1.594327874	0.00010761	up
101797964	THBS2	-1.739043988	2.62E-54	down
101801058	LOC101801058	-2.244614525	4.82E-48	down
101793107	GAS6	-1.04590389	3.65E-39	down
101793771	CDH11	-1.016637975	1.53E-37	down
101795323	VCAN	-1.653678117	7.76E-36	down
101803267	ST6GAL1	-1.591468903	1.60E-32	down
101796982	CYGB	-1.267548334	1.90E-32	down
101801406	CLMP	-1.130198944	1.11E-31	down
101794528	LOC101794528	-1.705774671	1.34E-31	down
101799982	FBLN5	-1.236717696	2.75E-31	down
101797642	CRISPLD2	-1.556351893	2.81E-31	down
101794665	RRM2	-1.127708905	2.39E-29	down
101790262	EDNRB	-1.29438353	6.05E-24	down
101805357	CRLF1	-1.754343048	2.05E-20	down
101796895	LRRC32	-1.78485848	6.02E-20	down
101800644	TSKU	-1.161517152	9.78E-19	down
101796320	HPGDS	-1.00593613	2.62E-18	down
101790805	FBN3	-1.112281952	5.71E-18	down
101791702	MXRA5	-2.307611821	1.94E-17	down
101804899	MGP	-3.831513827	3.97E-17	down

Table S2. The top twenty up-regulated and down-regulated genes between TG2 and TG3.

Gene ID	Gene name	Fold change	P value	
113840804	LOC113840804	2.492038883	5.58E-06	up
101795348	KCTD16	1.306369583	0.004408984	up
113843855	LOC113843855	4.745680436	0.005934833	up
113840902	LOC113840902	1.559494055	0.008629647	up
101794675	DZIP1L	1.962028308	0.013952311	up
113841713	LOC113841713	2.334473425	0.013955177	up
113844769	LOC113844769	4.559055111	0.014786018	up
110353918	LOC110353918	2.110189562	0.017058414	up
101790729	NPHS2	1.334113833	0.018852072	up
113843545	LOC113843545	1.418260686	0.020129638	up
106019440	LOC106019440	2.555993651	0.026485878	up
106015764	LOC106015764	3.661522721	0.029644143	up
101802032	ABLIM2	1.052919317	0.03039486	up
113843555	LOC113843555	4.15844347	0.030960463	up
106016887	LOC106016887	2.50272905	0.031513403	up
101797883	CCDC27	1.286957032	0.031529855	up
113840515	LOC113840515	3.503750307	0.031918004	up
113840436	LOC113840436	1.74165325	0.033210988	up
113843051	NKX2-2	2.508794285	0.034587345	up
106019320	LOC106019320	1.994936566	0.034871867	up
113841028	LOC113841028	-2.275726795	3.51E-58	down
101805147	SHH	-1.317796946	0.000142094	down
101790628	TTLL10	-2.514280294	0.000222744	down
101799156	TMEM173	-2.772247948	0.000761696	down
novel.72	-	-2.231776238	0.001707417	down
113845747	LOC113845747	-1.177005413	0.001722957	down
novel.303	-	-2.018081444	0.001738502	down
101793704	RNF186	-2.676382509	0.002134634	down
106014935	LOC106014935	-2.197459488	0.003150285	down
113843752	IRF7	-1.011419504	0.003662499	down
101790774	IL17C	-4.2958685	0.003931212	down
101800815	DUOXA2	-2.85439428	0.005000253	down
113843819	LOC113843819	-1.171578539	0.006609472	down
101803013	CCL20	-4.064026256	0.006958126	down
101804455	NOS2	-2.833672848	0.007321245	down
101794037	ACOD1	-4.989431592	0.008094668	down
113844326	CRIP1	-1.153490492	0.009307071	down
101798663	LOC101798663	-2.576321295	0.010116743	down
101804010	IL8	-3.00259321	0.010501104	down
101803817	LOC101803817	-2.670059205	0.012248534	down

Table S3. The top twenty up-regulated and down-regulated genes between CG2 and TG1.

Gene ID	Gene name	Fold change	P value	
101792965	RDH10	2.366519076	6.99E-247	up
113841577	LOC113841577	2.552219772	6.23E-246	up
101802788	COL3A1	1.321891409	8.76E-227	up
101794665	RRM2	2.448472324	1.26E-222	up
101797203	LOC101797203	1.398130766	1.33E-217	up
101805383	LGALS1	2.056729871	1.61E-200	up
101795751	CDC20	1.926482023	1.64E-184	up
101795528	CD81	1.326508086	7.47E-184	up
101797964	THBS2	2.647486076	8.31E-163	up
101797642	CRISPLD2	2.881525837	2.62E-162	up
101804915	DSP	1.439891311	2.66E-155	up
106015426	MAB21L2	1.763111613	1.53E-143	up
101804681	GM2A	1.404383535	2.63E-139	up
101804552	PLAU	1.084205927	4.49E-131	up
101794154	LIMK2	1.220510507	7.34E-129	up
101803169	CLEC3B	2.3054827	5.04E-124	up
101798901	ITGB4	1.988397166	1.45E-123	up
101789769	PTMS	1.161729259	3.98E-122	up
101793076	SYNPO2	2.765876683	1.16E-119	up
101794718	COL1A2	1.184986923	5.98E-119	up
101801633	SCIN	-1.625318366	3.68E-258	down
101790932	SQSTM1	-2.069890038	6.38E-232	down
101801232	LOC101801232	-1.887436695	1.35E-202	down
101793626	GEM	-2.063002929	3.45E-192	down
101790193	CCN2	-1.160424954	2.03E-189	down
101791516	LOC101791516	-1.377174813	2.23E-187	down
101799795	LOC101799795	-2.837838272	9.84E-183	down
101802378	LOC101802378	-1.628501465	9.09E-176	down
101796141	PDK4	-2.787247648	7.26E-174	down
101793120	ALAS1	-1.101160906	6.50E-173	down
113843349	LOC113843349	-2.031323703	9.33E-170	down
101792868	PLEKHO2	-1.917076	7.53E-168	down
101798273	OSGIN1	-2.446753013	4.18E-160	down
101790922	SOAT1	-1.286045698	2.00E-155	down
101799921	CREG1	-1.621135785	2.33E-155	down
101797741	CLDN1	-1.013561273	6.44E-155	down
101804450	ZFAND2A	-2.975677258	2.63E-154	down
110353167	LOC110353167	-3.13080782	4.53E-151	down
101801383	DIP2A	-1.35302783	9.97E-150	down
101791589	LPCAT3	-1.205610589	2.09E-128	down

Table S4. The top twenty up-regulated and down-regulated genes between CG2 and TG2.

Gene ID	Gene name	Fold change	P value	
101795751	CDC20	1.895647224	2.02E-284	up
101804915	DSP	1.06826909	2.03E-282	up
101792965	RDH10	1.927661572	7.71E-225	up
101796982	CYGB	1.638227936	3.30E-215	up
101790262	EDNRB	1.919220835	1.59E-201	up
106015426	MAB21L2	1.91953875	4.63E-197	up
101797969	LMNA	1.059370803	1.01E-162	up
101799544	SNAI1	1.808742989	3.60E-149	up
101798901	ITGB4	1.690665926	1.27E-131	up
101797569	LOC101797569	1.958114962	7.81E-128	up
101798848	PKP1	1.20417102	4.94E-123	up
101804681	GM2A	1.176455376	1.52E-119	up
101797285	PDGFRA	1.00871987	5.05E-117	up
101789702	GREM1	2.496127911	4.08E-112	up
101803029	CELSR1	1.560805666	5.71E-110	up
101799365	REM1	2.338496722	1.08E-107	up
101804534	ANPEP	1.716712177	2.04E-107	up
101789819	CAVIN1	1.268385645	9.54E-106	up
101803169	CLEC3B	1.444376422	9.55E-106	up
101792999	ZEB2	1.442180687	1.89E-105	up
101790193	CCN2	-1.01046	1.31E-280	down
101802003	TXNRD1	-1.96089	2.07E-276	down
101794153	TMEM45A	-1.9748	7.01E-266	down
101793376	IQSEC3	-2.38421	2.46E-255	down
101804310	TIMP3	-1.47887	1.76E-241	down
101804134	GPX3	-1.52519	2.39E-218	down
101790922	SOAT1	-1.19606	4.28E-207	down
101801834	ACSL5	-4.08909	8.98E-204	down
101799007	FADS1	-1.15897	4.94E-203	down
101796141	PDK4	-2.1805	1.83E-202	down
101799921	CREG1	-1.29364	1.44E-199	down
101804363	TMCC3	-1.37911	2.47E-185	down
101804465	LOC101804465	-3.20808	1.73E-178	down
101796319	CAPN5	-1.27929	8.91E-169	down
101802299	SULF1	-1.75342	3.25E-165	down
101792868	PLEKHO2	-1.6502	1.02E-163	down
101790932	SQSTM1	-1.16871	8.65E-162	down
101791467	LOC101791467	-3.08345	6.71E-161	down
101801232	LOC101801232	-1.65325	1.37E-157	down
101791589	LPCAT3	-1.03002	1.47E-155	down

Table S5. The top twenty up-regulated and down-regulated genes between CG2 and TG3.

Gene ID	Gene name	Fold change	P value	
101805383	LGALS1	1.973131221	1.18E-103	up
101795751	CDC20	1.840220885	1.39E-94	up
101797569	LOC101797569	1.941520743	7.63E-79	up
101793076	SYNPO2	2.447185601	6.77E-78	up
101803029	CELSR1	1.563044261	4.74E-75	up
101799365	REM1	2.403038704	1.27E-65	up
101795595	NPNT	1.938584941	3.95E-63	up
101792965	RDH10	1.962962227	2.53E-60	up
101792999	ZEB2	1.459017707	9.57E-59	up
101804534	ANPEP	1.75222347	6.38E-58	up
106015426	MAB21L2	2.064203416	1.08E-57	up
101795399	EFEMP1	1.739046331	6.34E-57	up
101804915	DSP	1.292248721	1.87E-54	up
101790262	EDNRB	1.797515683	1.01E-53	up
101789702	GREM1	2.410011306	5.34E-52	up
101792582	ROBO2	2.63922836	8.43E-47	up
101803169	CLEC3B	1.325207598	1.07E-44	up
101789769	PTMS	1.0174089	4.37E-43	up
101804752	VIL1	1.661906942	5.15E-41	up
novel.40	-	2.870598453	9.15E-41	up
101799743	ADAMTS15	-4.28338	6.43E-242	down
101792259	DHRS3	-2.9204	8.96E-236	down
113844003	LOC113844003	-3.58013	3.90E-184	down
101801349	SCD	-2.13694	4.41E-180	down
101796097	MUC13	-2.20511	1.89E-139	down
101804465	LOC101804465	-3.54929	1.54E-135	down
101793376	IQSEC3	-2.28568	1.27E-131	down
101801834	ACSL5	-3.88065	4.88E-129	down
101801633	SCIN	-1.76824	7.23E-129	down
101802003	TXNRD1	-1.9482	1.18E-128	down
101802063	TMEM86A	-1.75163	2.49E-119	down
101802378	LOC101802378	-2.05744	2.91E-114	down
101794153	TMEM45A	-2.1031	1.61E-109	down
101799752	LIPG	-2.56992	3.42E-90	down
101802299	SULF1	-1.79321	3.82E-88	down
113843349	LOC113843349	-1.83046	3.21E-81	down
101801232	LOC101801232	-1.56224	2.33E-77	down
101799921	CREG1	-1.40111	7.03E-74	down
101795897	CAPN6	-2.4258	1.72E-73	down
101792626	LOC101792626	-3.74444	8.11E-71	down
